# Biofilms and efflux pump regulatory gene (*mexR*) in multidrug-resistant *Pseudomonas aeruginosa* isolated from migratory birds in Egypt

**DOI:** 10.14202/vetworld.2022.2425-2431

**Published:** 2022-10-19

**Authors:** Esraa A. Elshafiee, Hanan S. Khalefa, Nayera M. Al-Atfeehy, Fatma Amer, Dalia A. Hamza, Zeinab S. Ahmed

**Affiliations:** 1 Department of Zoonoses, Faculty of Veterinary Medicine, Cairo University, P.O. Box 12211, Giza, Egypt; 2 Department of Veterinary Hygiene and Management, Faculty of Veterinary Medicine, Cairo University, P.O. Box 12211, Giza, Egypt; 3 Biotechnology Unit, Reference Laboratory for Veterinary Quality Control on Poultry Production (RLQP), Animal Health Research Institute (AHRI), Agriculture Research Center (ARC), Nadi El-Seid St., P.O. Box 246, Dokki, Giza, 12618, Egypt; 4 Biotechnology Unit, Animal Health Research Institute, Animal Research Center, Nadi El-Seid St., P.O. Box 246, Dokki, Giza, 12618, Egypt

**Keywords:** biofilm, Egypt, *mexR*, migratory birds, multidrug-resistant, *Pseudomonas aeruginosa*

## Abstract

**Background and Aim:**

Multidrug-resistant (MDR) *Pseudomonas aeruginosa* is a global threat to public health. This study aimed to determine biofilms and efflux pump regulatory gene (*mexR*) in MDR *P. aeruginosa* isolates.

**Materials and Methods:**

A total of 42 fecal samples of aquatic migratory birds collected during hunting season in Egypt were evaluated for the detection of *P. aeruginosa* according to standard culture-based methods. The antibiotic susceptibility of *P. aeruginosa* strains was evaluated using disk diffusion methods. The biofilm formation ability of the isolates was phenotypically determined using a colorimetric microtitration plate assay. Polymerase chain reaction amplification was performed to detect biofilm genes (*PelA* and *PslA*) and *mexR*.

**Results:**

In total, 19 isolates (45.2%) were recovered from the 42 fecal samples of migratory birds. All isolates were identified as MDR *P. aeruginosa*, and 78.9% of the strains produced biofilms at different degrees. Molecular detection of biofilm extracellular polymeric substances revealed that *PelA* was the most predominant gene in the biofilm-producing isolates, followed by *PslA*. *mexR* was detected in 63.2% of MDR *P. aeruginosa* isolates, and its prevalence was higher in non–biofilm-producing strains (75%) than in biofilm-producing strains (60%).

**Conclusion:**

Antibiotic resistance in *P. aeruginosa* isolates recovered from migratory birds through various mechanisms is a major public and animal health problem. It is important to consider the significance of migratory birds in disease transmission.

## Introduction

The World Health Organization has recognized antibiotic resistance as a serious global crisis not only in terms of human health but also for animals (both domestic and wildlife) and the environment [1]. Zoonotic bacterial strains from wild birds can be transmitted to humans and animals through the environment, such as through feces-polluted water, soil, food habits of wild animals/birds, meat, or direct contact during human hunting. Moreover, due to their eating habits, long-distance migration, and habitation in a range of settings, migratory birds are increasingly susceptible to infection with antibiotic-resistant bacteria, particularly in places where people live [2]. Consequently, these birds have historically played a vital role as carriers of pathogenic bacteria and reservoirs for drug-resistant bacteria, a concern that has been receiving attention in recent years [3–5].

Among the opportunistic gram-negative bacteria, *Pseudomonas aeruginosa* is the primary cause of nosocomial and life-threatening infections in immunocompromised patients [6]. *Pseudomonas aeruginosa* also plays a pivotal role in progressive lung destruction and subsequent respiratory failure in patients with cystic fibrosis and is thus responsible for increasing the rates of hospitalization and mortality [7]. The ability of this bacterium to acquire antibiotic resistance is a major issue in terms of preventing and/or treating the infections it causes [8].

In general, the major mechanisms of antibiotic resistance in *P. aeruginosa* can be classified into intrinsic, acquired, and adaptive. The innate resistance of *P. aeruginosa* involves its low outer membrane permeability and the overexpressed efflux pump that ejects antibiotics from the cell [9]. The acquired resistance of *P. aeruginosa* could be attained by the acquisition of resistance genes through horizontal transfer or mutational alterations [10]. Biofilm formation is considered an adaptive resistance of *P. aeruginosa*, serving as a diffusion barrier to limit the entry of antibiotics into the bacterial cell [11, 12].

Biofilms are complex bacterial communities embedded in a matrix of extracellular polymeric substances (EPS), including proteins, extracellular DNA, and exopolysaccharides. The exopolysaccharide component of the biofilm matrix can inhibit the penetration of antibiotics and resist phagocytosis by host immune cells [13]. Genetically, most *P. aeruginosa* strains can synthesize three exopolysaccharides, *Pel*, *Psl*, and alginate [14]. Biofilms are also important in the spread of antibiotic resistance by horizontal gene transfer [15].

Efflux pump systems comprise a primary mechanism in *P. aeruginosa* that confers multidrug resistance. MexAB–OprM is one of the largest multidrug-resistant (MDR) efflux pump systems with high expression levels in *P. aeruginosa* [8].

The expression of MexAB–OprM is regulated by *mexR* [16]. MexR is a 147-amino-acid protein that suppresses the production of MexAB–OprM [17]. When bacteria with a mutant *MexR* gene are exposed to a specific antibiotic, the production of *MexA*, *MexB*, and *OprM* proteins is increased, leading to the hyperexpression of efflux pumps and increased antibiotic resistance [8, 18]. Several independent studies have shown that overexpression of efflux pumps contributes to the multidrug resistance phenotype [19, 20].

There is limited knowledge of antibiotic resistance mechanisms in *P. aeruginosa* isolated from migratory birds in Egypt. Therefore, this study aimed to characterize the biofilm formation ability and the presence of the efflux pump regulatory gene (*mexR*) in MDR *P. aeruginosa* isolated from different species of migratory birds.

## Materials and Methods

### Ethical approval

The animal study was reviewed and approved by the Institutional Animal Care and Use Committee of the Faculty of Veterinary Medicine, Cairo University (VetCU20022020141). All methods were carried out in accordance with relevant guidelines and regulations. There were no experiments on human participants.

### Study period and location

The study was conducted from September 2017 to December 2018 in two distinct geographic regions at El-Fayoum and Port Said Governorate in Egypt.

### Bird collection

A total of 42 apparently healthy live migratory aquatic birds (Table-1) were collected in Egypt from two different geographic locations across the shore of the Mediterranean Sea at El-Fayoum and Port Said during hunting season. All birds were trapped live using nets. After trapping, cloacal swabs were collected in tubes containing 2 mL of sterile saline (0.9% NaCl) and stored in an icebox until transported to the laboratory. The birds were set free immediately after collecting the biological material.

**Table-1:** Type and number of migratory birds collected.

English name	Binomial name	Location	Status	No. of birds
Northern Shoveler	*Anas Clypeata*	Port-said El-Fayoum	Passage migrant and winter visitor	14
Green-Winged Teal	*Anas Carolinensis*	El- Fayoum	Passage migrant and winter visitor	13
Eurasian Coot	*Fulica Atra*	Port-said	Passage migrant and winter visitor	10
Mallard	*Anas platyrhynchos*	El- Fayoum	Passage migrant and winter visitor	5

### Isolation and identification of *P. aeruginosa*

On arrival at the laboratory, the fecal samples were inoculated into tryptic soy broth and incubated at 37°C for 24 h. Next, a loopful of previously inoculated broths was cultured on *Pseudomonas* agar base medium with C-N supplement (Oxoid, Hampshire, UK) and incubated at 37°C for 48 h. Subculture on nutrient agar plates was also done for further identification.

### Antibiotic susceptibility testing of *P. aeruginosa*

The antimicrobial sensitivity profile of the recovered *P. aeruginosa* isolates was determined using the disk diffusion assay on Muller–Hinton agar (Oxoid) according to the standards and interpretive criteria described by the Clinical and Laboratory Standards Institute (CLSI) guidelines [21]. The strains were evaluated for susceptibility to the following panel of antibiotics (Oxoid): Enrofloxacin (ENR) 5 μg, penicillin (P) 10 μg, lincomycin (L) 2 μg, tetracycline (TE) 30 μg, trimethoprim (TR) 5 μg, neomycin (N) 30 μg, chloramphenicol 30 μg, gentamicin (CN) 10 μg, erythromycin (E) 15 μg, and ceftriaxone (CRO) 30 μg. Based on the diameter of the zones of inhibition, the isolates were classified as resistant, intermediate, or susceptible according to the CLSI guidelines [21].

### Biofilm formation

Biofilm quantification assay was performed in 96-well polystyrene microplates as described previously [22]. Briefly, 200 µL of each tested *P. aeruginosa* isolate (cultured in BHI broth at 37°C for 24 h) was added in triplicate in 96-well flat-bottomed plastic microtiter plates with a lid. The polystyrene microplates were incubated at 37°C for 24 h, after which the bacterial suspensions in each well were removed and washed three times with 250 µL of sterile saline solution (0.9% NaCl). Each well was fixed with 200 µL of methanol for 15 min. Once fixed, the methanol was slowly removed, and then the plates were left to dry at room temperature (30°C). Subsequently, the wells were stained with 200 µL of crystal violet solution for 5 min, washed with running water, and then left to dry at 30°C. The evaluation of the biofilm formation ability depends on the absorbance readings taken in an enzyme-linked immunosorbent assay reader (BioRad, model 550, Srl, Italy) at a wavelength of 570 nm, then classified according to Stepanović *et al.*, [22]. BHI broth without the bacterial inoculum was used as the negative control, and the optical density (OD) for each isolate (ODi) was obtained by averaging the values of three wells, which was compared with the OD of the negative control (ODc). The results were assorted into the following four categories according to the mean OD in relation to the negative control result (ODc): nonadherent (−) if ODi ≤ ODc; weakly adherent (+) if ODc < ODi ≤ 2 × ODc; moderately adherent (++) if 2 × ODc < ODi ≤ 4 × ODc; and strongly adherent (+++) if 4 × ODc < ODi.

### Molecular identification of *P. aeruginosa* isolates

All isolates obtained from the examined samples were genotyped using a polymerase chain reaction (PCR) assay.

PCR amplification of biofilm genes (*PelA* and *PslA*) and the efflux pump regulatory gene was performed on positive *P. aeruginosa* isolates (19 isolates).

DNA extraction was performed using the QIAamp DNA mini kit according to the manufacturer’s instructions (Qiagen, Germany, GmbH). Table-2 shows the primer sequence, annealing temperature, and the size of the amplified product [23–25].

**Table-2:** Oligonucleotide primers for detection of *Pseudomonas aeruginosa*, biofilm genes (*PelA, PslA*), and efflux pump regulatory gene (*mexR*).

Target	Gene	Sequence 5’-3’	Annealing temp (°C)	Reference	Amplified fragment
*Pseudomonas* species	16S rDNA	GACGGGTGAGTAATGCCTA CACTGGTGTTCCTTCCTATA	54	[21]	618
*Pseudomonas aeruginosa*	16S rDNA	GGGGGATCTTCGGACCTCA TCCTTAGAGTGCCCACCCG	58		956
Biofilm genes	*PelA*	CATACCTTCAGCCATCCGTTCTTC CGCATTCGCCGCACTCAG	60	[22]	786
	*PslA*	TCCCTACCTCAGCAGCAAGC TGTTGTAGCCGTAGCGTTTCTG	60		656
Efflux pump regulatory gene	*MexR*	GCGCCATGGCCCATATTCAG GGCATTCGCCAGTAAGCGG	57	[23]	673

PCR amplification was performed in a 25-µL reaction mixture containing 12.5 µL of Emerald Amp. Max PCR Master Mix (Emerald, Japan), 1 µL of each primer (20 pmol concentration), 4.5 µL of nuclease-free water, and 6 µL of a template in a Biometra T3 thermal cycler. The PCR products were separated by electrophoresis on a 1.5% agarose gel stained with ethidium bromide. The gel was photographed using Alpha Innotech gel documentation system (Biometra GmbH, Gottingen, Germany).

## Results

### Isolation and identification of *P. aeruginosa*

A total of 19 isolates (45.2%) were recovered from the fecal samples of 42 migratory birds. Specific colonies, which produced the characteristic growth features of *P. aeruginosa* on specific media, were confirmed by identifying 16sDNA genes.

### Antimicrobial susceptibility testing

The susceptibility profile against ten antibiotics belonging to different categories was evaluated. All *P. aeruginosa* isolates were highly resistant (100%) to L and E followed by P (94.7%). Moreover, they exhibited the same level of resistance (89.5%) to ENR, oxytetracycline, and TR. All isolates were susceptible (100%) to CRO, and 94.7% and 89.5% of the isolates were susceptible to CN and N, respectively (Table-3). The antibiotic resistance pattern of all *P. aeruginosa* isolates (19 isolates) is shown in Table-4.

**Table-3:** The susceptibility profile of *Pseudomonas aeruginosa* isolates from migratory birds against ten different antibiotics.

Antibiotic	No. (%) of resistant	No. (%) of sensitive
ENR 5 μg	17 (89.5)	2 (10.5)
P 10 μg	18 (94.7)	1 (5.3)
L 2 μg	19 (100)	0 (0)
TE 30 μg	17 (89.5)	2 (10.5)
TR 5 μg	17 (89.5)	2 (10.5)
N 30 μg	2 (10.5)	17 (89.5)
C 30 μg	8 (42.1)	9 (47.4)
CN 10	1 (5.3)	18 (94.7)
E 15 μg	19 (100)	0 (0)
CRO 30 μg	0 (0)	19 (100)

ENR=Enrofloxacin, P=Penicillin, L=Lincomycin, TE=Tetracyclin, TR=Trimethoprim, N=Neomycin, C=Chloramphenicol, CN=Gentamicin, E=Erythromycin, CRO=Ceftriaxone

**Table-4:** Antibiotic resistance pattern of 19 *Pseudomonas aeruginosa* isolates.

Isolate	Antibiotic resistance	Resistance pattern
P-1	ENR, P, L, TR, TE, N, C, E	MDR
P-2	ENR, P, L, TR, TE, N, E	MDR
P-3	ENR, P, L, TR, TE, E	MDR
P-4	ENR, P, L, TR, TE, E	MDR
P-5	P, L, E	MDR
P-6	ENR, P, L, TR, TE, C, E	MDR
P-7	ENR, L, TR, TE, CN, C, E	MDR
P-8	ENR, P, L, TR, TE, E	MDR
P-9	ENR, P, L, TR, TE, C, E	MDR
P-10	ENR, P, L, TR, TE, C, E	MDR
P-11	ENR, P, L, TR, TE, C, E	MDR
P-12	ENR, P, L, TR, TE, C, E	MDR
P-13	ENR, P, L, TR, TE, E	MDR
P-14	P, L, TR, TE, E	MDR
P-15	ENR, P, L, TR, TE, E	MDR
P-16	ENR, P, L, TR, TE, E	MDR
P-17	ENR, P, L, E	MDR
P-18	ENR, P, L, TR, TE, E	MDR
P-19	ENR, P, L, TR, TE, E	MDR

ENR=Enrofloxacin, P=Penicillin, L=Lincomycin, TE=Tetracyclin, TR=Trimethoprim, N=Neomycin, C=Chloramphenicol, CN=Gentamicin, E=Erythromycin, CRO=Ceftriaxone, MDR=Multidrug-resistant

### Biofilm production and genetic basis of biofilm formation

In the phenotypic test, considered as the “gold standard” for biofilm detection, 78.9% (15/19) of the isolates were identified as biofilm producers. These isolates produced biofilms at different degrees and were categorized as follows: About 26.3% (5/19) were weak biofilm producers, 21% (4/19) were moderate biofilm producers, and 31% (6/19) were strong biofilm producers (Table-5). The presence of *Pel A* and *PslA* in the isolates was analyzed by PCR. *PelA* was the most predominant gene, followed by *PslA* (Table-5).

**Table-5:** Phenotypic and genotypic biofilm profile of 19 MDR *Pseudomonas aeruginosa* isolates.

Biofilm profile	No. of isolates (%) Total (n = 19)	Biofilm genes	

		*PelA*	*PslA*
Non-biofilm producer (-)	4 (21)	1	-
Weak biofilm producer (+)	5 (26.3)	2	-
Moderate biofilm producer (++)	4 (21)	1	1
Strong biofilm producer (+++)	6 (31)	4	1
Total biofilm producer	15 (78.9)	7	2

MDR=Multidrug-resistant

### Efflux pump regulatory gene (*mexR*)

The presence of the efflux pump regulatory gene (*mexR*) was recorded in 63.2% of MDR *P. aeruginosa* isolates. The prevalence of *mexR* was higher in non-biofilm producers (75%) than in biofilm producer strains (60%) (Table-6).

**Table-6:** Occurrence of efflux pump regulatory gene (*mex R*) in biofilm producing and non-biofilm producing MDR *Pseudomonas aeruginosa* isolates.

Gene	Total (n = 19)	No. of isolates (%)	

		Biofilm producer (n = 15)	Non-biofilm producer (n = 4)
*MexR* (+)	12 (63.2%)	9 (60%)	3 (75%)

MDR=Multidrug-resistant

## Discussion

A bacteriological examination of the fecal samples of migratory birds revealed the presence of *P. aeruginosa* with a prevalence of 45.2%. This result was much higher than that reported by Ahmed *et al*. [5], who detected 18.3% of *P. aeruginosa* isolates in migratory birds in Egypt. This finding confirms that migratory birds may harbor highly pathogenic bacteria that can infect humans directly or indirectly through environmental dissemination. Therefore, it is essential to consider the potential role of migratory birds in transmitting such pathogens.

The antibiogram of *P. aeruginosa* isolates demonstrated high resistance to L and E, followed by P. Furthermore, the isolates exhibited the same resistance level (89.5%) to ENR, oxytetracycline, and TR. Compared to our results, a previous study reported a high resistance level to CRO in *P. aeruginosa* isolated from camels in Egypt [26].

Phenotypically, all *P. aeruginosa* isolates examined in the present study exhibited resistance to multiple antibiotics. The presence of MDR bacteria in migratory birds indicates a public health concern due to the increased prevalence of zoonotic diseases [27]. However, scientists have speculated that migratory animals, especially migratory birds, carry resistant bacteria or genes and transport them to regions far from anthropogenic influences [2].

Although bacterial biofilms play a critical role in a variety of infectious diseases, they are frequently overlooked. To the best of our knowledge, this is the first study to determine the biofilm formation ability of MDR *P. aeruginosa* in migratory birds. The microtitration test revealed that 78.9% of the isolates produced biofilms, of which 26.3% were weak biofilm producers, 21% were moderate biofilm producers, and 31% were strong biofilm producers. In a previous study by Jabalameli *et al*. [28], >96% of *P. aeruginosa* isolates recovered from patients with burn injuries were found to produce biofilms, of which 22.9% were weak biofilm producers, 26% were moderate biofilm producers, and 47% were strong biofilm producers.

The relative importance of the EPS matrix in *P. aeruginosa* biofilms is dependent on the genetic background of strains, nutritional conditions, and developmental phases of biofilms [29]. At least three polysaccharides (*Psl*, *Pel*, and alginate) have been identified in *P. aeruginosa* that play key roles in structure maintenance and antibiotic resistance of the biofilm [30].

Molecular identification of EPS genes showed that *PelA* was the most predominant gene in biofilm producers, followed by *PslA* (Table-5). However, other studies have reported a higher incidence of *pslA* [31, 32].

Our important finding was that the increase in antibiotic resistance occurred independently of the amount of biofilm produced, which has been supported by Qi *et al*. [33], who discovered that despite individual differences among isolates, the antibiotic resistance, biofilm formation ability, and biofilm-specific resistance are all linked. These genetic findings support our results that biofilm-specific resistance can be regulated irrespective of biofilm quantity.

The findings of our study indicate a significant public health concern because the migratory birds could disseminate *P. aeruginosa* strains possessing the ability to produce biofilms, which constitute a significant virulence factor in several nosocomial infections [34]. Furthermore, environmental reservoirs of antibiotic-resistant *P. aeruginosa* near hospitals have been documented by Elshafiee *et al*. [35] and Cherak *et al*. [36], who emphasized the pivotal role of contaminated water in the transmission of such stains to humans and animals.

Efflux pumps allow bacterial cells to pump out intracellular toxins, including antibiotic drugs. The MexAB–OprM efflux pump is one of the largest MDR efflux pumps with high expression levels in *P. aeruginosa*, which is controlled by the regulatory gene *mexR* [37]. In the present study, 63.2% of MDR *P. aeruginosa* isolates harbored *mexR*, which is an alarming finding because of the possibility of *mexR* mutations, which in turn will activate the mexAB–oprM operon and increase resistance to a range of different antibiotics [38]. Consequently, hyperexpression of mexAB–oprM has been detected in MDR clinical isolates resulting from mutations acquired in the repressor gene *mexR* [39]. This problem needs further investigation to detect the point mutation.

The prevalence of *mexR* was higher in non-biofilm producer strains than in biofilm producer strains. This finding is consistent with a previous study [40] that showed that the expression of MDR pumps is not increased in *P. aeruginosa* biofilms, implying that the innate antibiotic resistance in this bacterial population is due to other causes such as decreased membrane permeability and/or altered antimicrobial targets. In fact, biofilm-specific resistance is predominantly influenced by the organism’s level of antibiotic resistance, which may primarily cause augmentation [41].

Considering that wildlife isolates do not contact directly with antibiotics, the resistance observed among the investigated strains is alarming. This is largely attributed to the overuse of antibiotics in humans and veterinary medicine, resulting in the environmental dissemination of resistance genes.

## Conclusion

The present levels of bacterial resistance to an antibiotic in migratory birds indicate a threat to public and animal health. Analysis of the phenotypic and genotypic resistance profiles of the *P. aeruginosa* isolates revealed their multidrug resistance characteristic. The isolates also exhibited the ability of biofilm formation which makes them more resistant to the action of antimicrobial agents. The efflux pump regulatory gene *mexR* showed a high prevalence in the MDR *P. aeruginosa* isolates, which may be responsible for controlling the expression of mexAB–oprM and its impact on the resistance toward a range of different antibiotics. There is an urgent need for control measures to limit the continuous abuse of antibiotics, especially in veterinary medicine, to avoid the environmental dissemination of antibiotic-resistant bacteria that form a possible link between animals, humans, and wildlife. A limitation of this study is the relatively small sample size of the wild birds. So, future studies should be based on a large sample size.

## Data Availability

All data generated or analyzed during this study are included in this published article.

## Authors’ Contributions

EAE, HSK, and ZSA: Designed the study, performed the methodology, investigation, and drafted the manuscript. NMA, FA and DAH: Performed methodology and reviewed and edited the manuscript. All authors have read and approved the final manuscript.
